# Microarray profiling and co-expression network analysis of lncRNAs and mRNAs in ovarian cancer

**DOI:** 10.1038/s41420-019-0173-7

**Published:** 2019-05-07

**Authors:** Ce Gao, Di Zhao, Qing Zhao, Dandan Dong, Lin Mu, Xuejun Zhao, Man Guo, Aili Xu, Lei Fang, Qian Liu, Jianhua Che

**Affiliations:** Department of Obstetrics and Gynecology, The Second Affiliated Hospital of Harbin Medical University, Harbin, China

**Keywords:** Tumour biomarkers, Ovarian cancer

## Abstract

Dysregulated long noncoding RNAs (lncRNAs) are involved in the pathogenesis and development of human diseases, such as epithelial ovarian cancer (EOC). In this study, we identified EOC-related lncRNAs and performed lncRNA and mRNA microarray analyses using IOSE80, a normal ovary cell line, and two ovarian carcinoma cell lines (SKOV3 and SKOV3/DDP) to investigate the potential roles of lncRNAs in EOC. lncRNA-HEIH expression in EOC tissues and cell lines was measured by quantitative real-time polymerase chain reaction (qPCR). In addition, we generated a lncRNA–mRNA co-expression network in order to identify lncRNA-expression trends and potential lncRNA target genes. Cell viability, migration, and invasion were determined by Cell Counting Kit-8, transwell assay, and wound-healing assay, respectively, and apoptosis was analyzed by flow cytometry. We identified 3527 differentially expressed lncRNAs upon comparison of the lncRNA profiles from IOSE80 with those of SKOV3 cell lines, with 11 differentially expressed lncRNAs confirmed by qPCR. Both pathway and gene ontology analyses demonstrated the involvement of lncRNAs, especially *HEIH* and *LINC-PINT*, in multiple biological processes. Furthermore, in vitro knockdown experiments confirmed that suppression of *HEIH* expression inhibited EOC cell proliferation. Our findings provide a foundation for further research into the role of these lncRNAs in EOC carcinogenesis and progression.

## Introduction

Ovarian cancer (OC) is the third most common cancer in women and the most fatal gynecological cancer in the world. According to a recent study, 22,280 new cases of OC and 15,500 OC-related deaths have been reported in the United States^1^. Epithelial ovarian cancer (EOC) constitutes 90% of all OC cases and is typically diagnosed at an advanced stage due to rapid progression of the disease and nonspecific clinical symptoms. Despite recent progress in surgery and chemotherapy, the overall 5-year survival rate of EOC patients is only 40%^2^. Due to the poor understanding of EOC etiology and pathogenesis, the molecular mechanisms associated with EOC progression have not been clearly elucidated, which is critical for the development of new therapeutic and diagnostic approaches.

Long noncoding RNAs (lncRNAs) are nonprotein-coding RNAs of >200 nucleotides and involved in the regulation of gene expression^3^. lncRNA-expression levels exhibit high degrees of tissue and disease specificity, which are closely associated with their biological functions^4^. Recent studies show that dysregulated lncRNAs play vital roles in tumorigenesis^5^ via multiple cancer-related biological processes, including apoptosis, cell cycle regulation, metastasis, and DNA-damage response^6–8^. Several lncRNAs, such as *HOTAIR*^9^, *FAL1*^10^, and *HOST2*^11^, are involved in EOC progression, with *ZFAS1* upregulated by cisplatin in multiple EOC cell lines^12^. In addition, a peptide nucleic acid-based therapeutic approach decreased EOC invasiveness and increased chemotherapeutic sensitivity by inhibiting *HOTAIR*–*EZH2* activity^13^. Moreover, the lncRNA *FAL1* regulates cancer-cell cycle progression and cell senescence, as well as xenograft tumor growth in vivo^14^, and another study reported that inhibiting the expression of lncRNA *HOST2* significantly reduced the migration, invasion, and proliferation of OVCAR-3 cells^15^. However, the regulatory roles of lncRNAs in EOC have not been studied sufficiently.

In this study, we performed genome-wide lncRNA and mRNA microarray analyses on IOSE80 cells, a normal ovary cell line, and two OC cell lines (SKOV3 and SKOV3/DDP) in order to identify EOC-related lncRNAs. The SKOV3 cell line is a human OC adenocarcinoma cell line, and SKOV3/DDP is a cisplatin-resistant variant of SKOV3^16^. Our findings revealed functional lncRNA profiles in EOC and *cis*-resistant EOC cells, thereby expanding the current understanding of EOC pathogenesis and providing novel insights supporting the development of new therapeutic targets for EOC.

## Results

### Determination of lncRNA-expression profiles in different cell lines

**Fig. 1:**
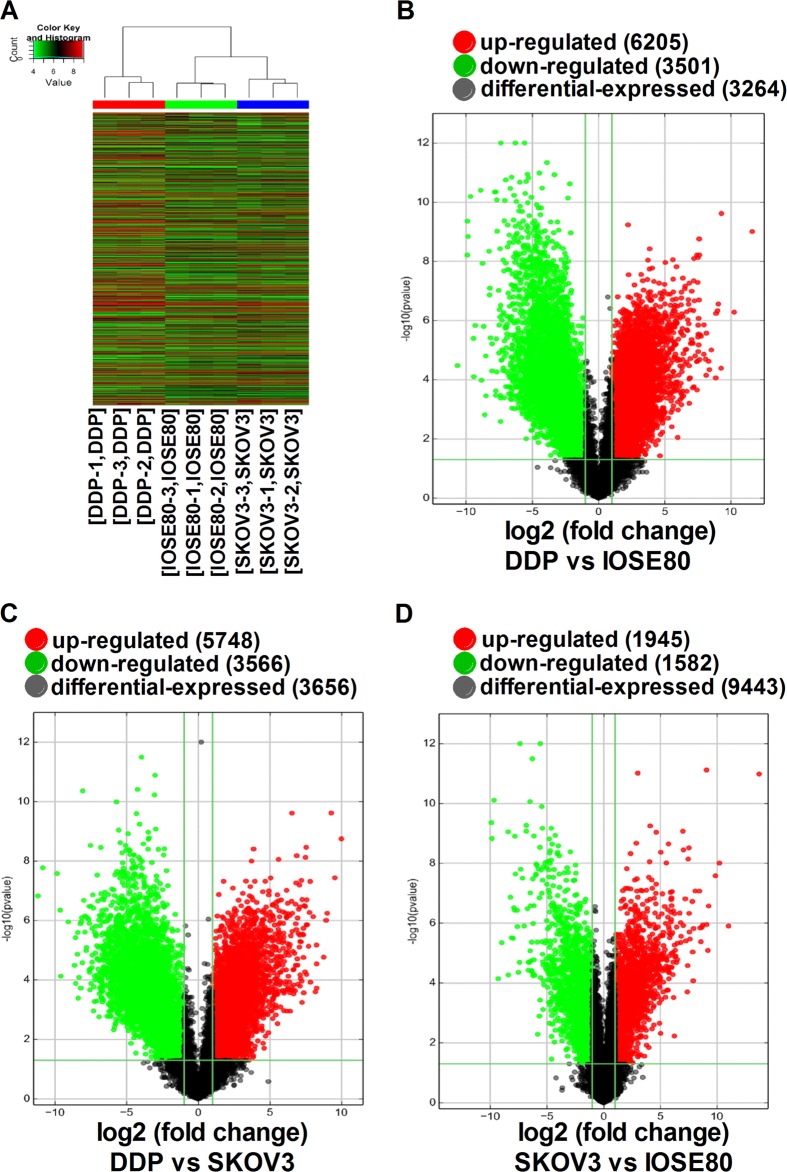
Differentially expressed lncRNAs in IOSE80, SKOV3, and SKOV3/DDP cell lines. **a** Differentially expressed lncRNAs were analyzed using hierarchical clustering. ‘Red’ indicates high relative expression, and ‘green’ indicates low relative expression. Volcano plot comparisons of gene expression between **b** SKOV3 and IOSE80 cells, **c** SKOV3/DDP and IOSE80 cells, and **d** SKOV3/DDP and SKOV3 cells. Vertical lines correspond to twofold upregulation and downregulation, respectively, and the horizontal line represents a *P* < 0.05. The red and green points in the plot represent upregulated and downregulated genes that are statistically significant

We profiled IOSE80, SKOV3, and SKOV3/DDP cell lines in order to determine transcriptional alterations. A total of 40,173 lncRNAs were analyzed using the Arraystar human lncRNA microarray version 4.0, and differences in lncRNA-expression levels according to fold changes >2.00 and a *P* < 0.05 were considered significant. Hierarchical clustering showed distinguishable lncRNA-expression profiles among the different cell lines, with the results indicating 3527 differentially expressed lncRNAs between SKOV3 and IOSE80 cells, of which 1945 were upregulated and 1582 were downregulated. A total of 9706 differentially expressed lncRNAs were identified in the SKOV3/DDP cell line as compared with the IOSE80 cell line and included 6205 upregulated and 3501 downregulated lncRNAs. Moreover, the expression profiles of 9314 lncRNAs differed between SKOV3/DDP and SKOV3 cells, including 5748 upregulated and 3566 downregulated lncRNAs. Figure 1 shows volcano plots and hierarchical clustering of the differentially expressed lncRNAs.

### mRNA profiles of the different cell lines

**Fig. 2:**
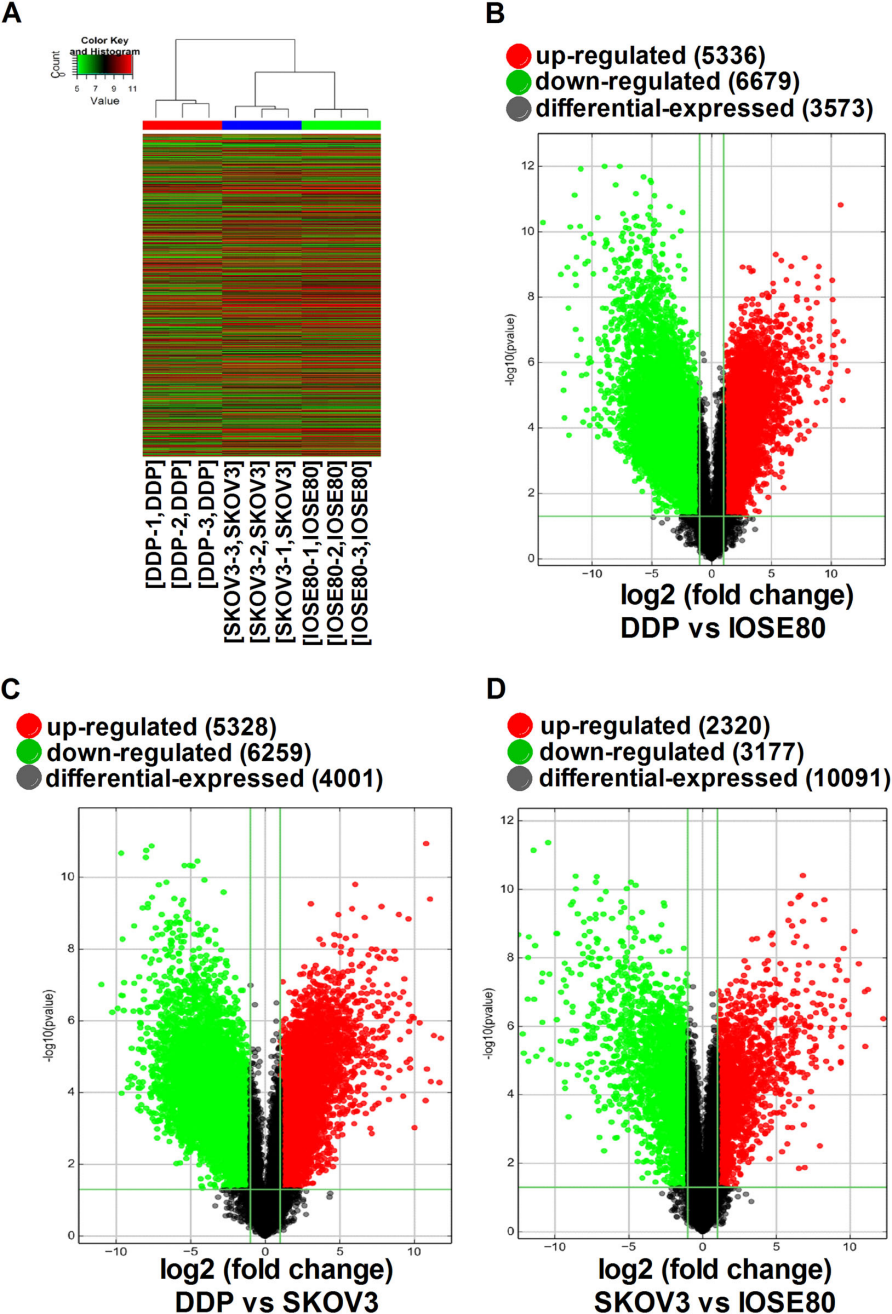
Differentially expressed mRNAs in IOSE80, SKOV3, and SKOV3/DDP cell lines. **a** Differentially expressed mRNAs were analyzed using hierarchical clustering. Volcano plot comparisons of gene expression between **b** SKOV3 and IOSE80 cells, **c** SKOV3/DDP and IOSE80 cells, and **d** SKOV3/DDP and SKOV3 cells. Volcano plots were used to visualize differential expression between two different conditions. Vertical lines correspond to twofold upregulation and downregulation, respectively, and the horizontal line represents a *P* < 0.05. The red and green points in the plot represent upregulated and downregulated genes that are statistically significant

We performed microarray profiling of mRNAs, identifying a total of 5497 differentially expressed mRNAs between SKOV3 and IOSE80 cells, including 2320 upregulated and 3177 downregulated mRNAs. In addition, 12,015 mRNAs were differentially expressed between SKOV3/DDP and IOSE80 cells, including 5336 upregulated and 6679 downregulated mRNAs. Moreover, we identified 11,587 differentially expressed mRNAs between SKOV3/DDP and SKOV3 cells, including 5328 upregulated and 6259 downregulated mRNAs. Figure 2 shows volcano plots and hierarchical clustering results for the differentially expressed mRNAs.

### Biological analysis

**Fig. 3:**
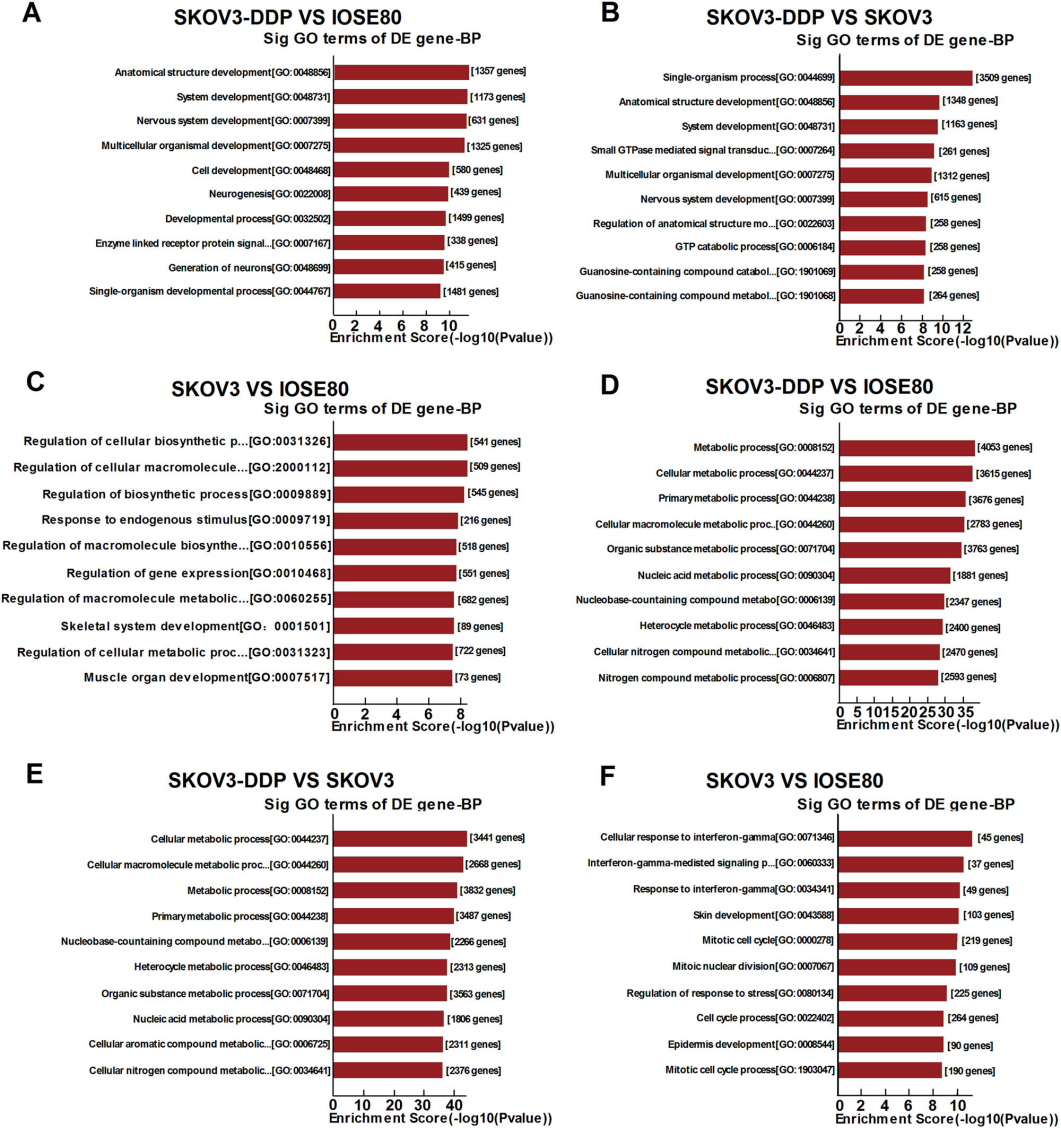
GO analysis of differentially expressed mRNAs. **a**–**c** Top ten GO terms of biological processes for mRNAs downregulated between SKOV3 and IOSE80 cells, SKOV3/DDP and IOSE80 cells, and SKOV3/DDP and SKOV3 cells. **d**–**f** Top ten GO terms of biological processes for mRNAs upregulated between SKOV3 and IOSE80 cells, SKOV3/DDP and IOSE80 cells, and SKOV3/DDP and SKOV3 cells

In our survey of gene ontology (GO) biological processes, the functions of the upregulated mRNAs (SKOV3 versus IOSE80, SKOV3/DDP versus IOSE80, and SKOV3/DDP versus SKOV3) were primarily involved in anatomical structure development, single-organism processes, and regulation of cellular biosynthetic processes (Fig. 3a–c). In addition, the functions of the downregulated mRNAs primarily involved cellular metabolic processes, cellular macromolecule metabolic processes, and cellular response to interferon (IFN)-γ (Fig. 3d–f).

**Fig. 4:**
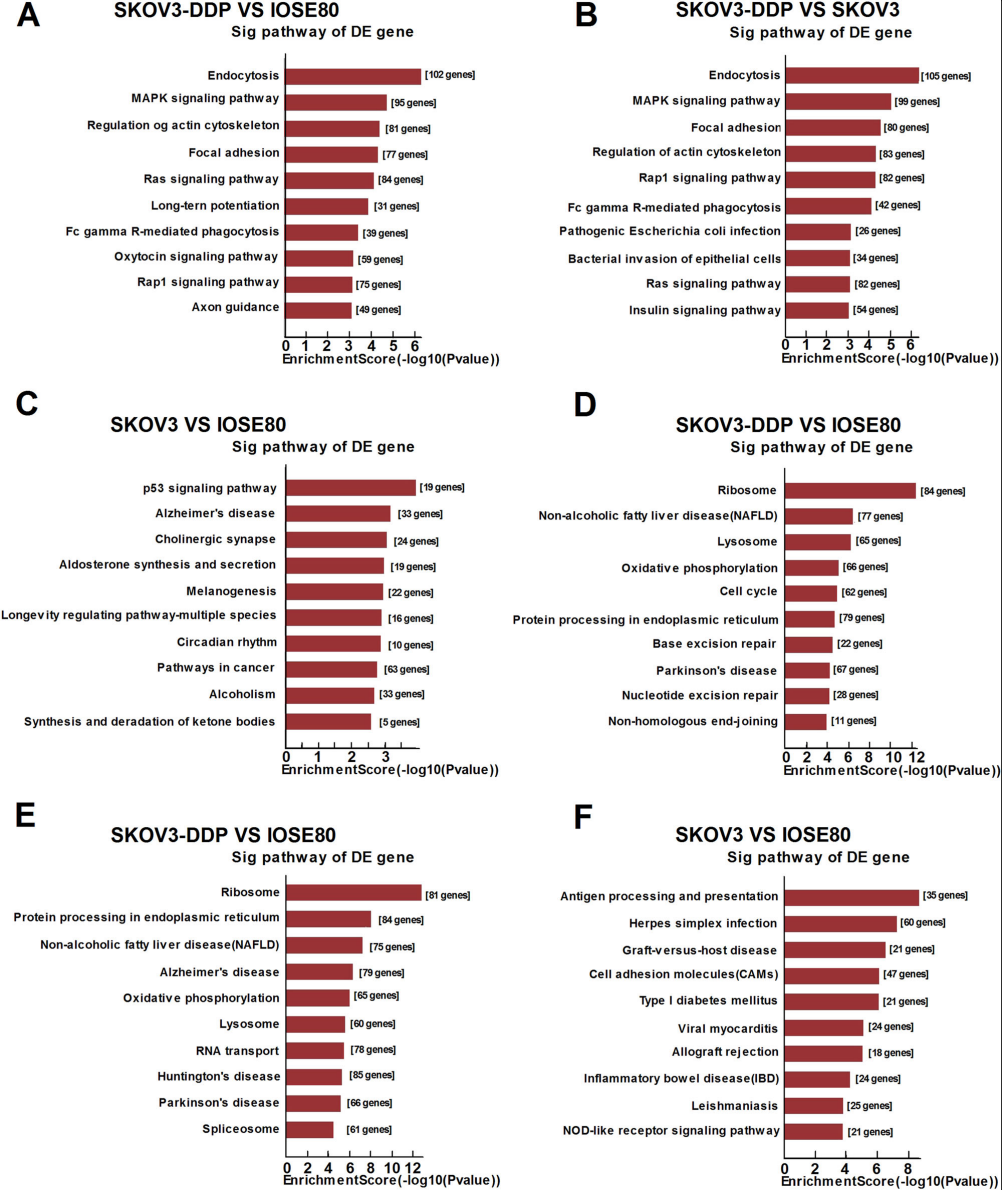
Pathway analysis of differentially expressed mRNAs. **a**–**c** Top ten KEGG pathways corresponding to the mRNAs downregulated between SKOV3 and IOSE80 cells, SKOV3/DDP and IOSE80 cells, and SKOV3/DDP and SKOV3 cells. **d**–**f** Top ten KEGG pathways corresponding to the mRNAs upregulated between SKOV3 and IOSE80 cells, SKOV3/DDP and IOSE80 cells, and SKOV3/DDP and SKOV3 cells

The main Kyoto encyclopedia of genes and genomes (KEGG) pathways for the upregulated transcripts (SKOV3/DDP versus IOSE80, SKOV3/DDP versus SKOV3, and SKOV3 versus IOSE80) involved endocytosis, the mitogen-activated protein kinase (MAPK) signaling pathway, and the p53 signaling pathway (Fig. 4a–c). By contrast, the main KEGG pathways for the downregulated transcripts included ribosome, protein processing in the endoplasmic reticulum, antigen processing and presentation, and herpes simplex infection (Fig. 4d–f).

### Candidate lncRNA-expression levels in vivo

**Fig. 5:**
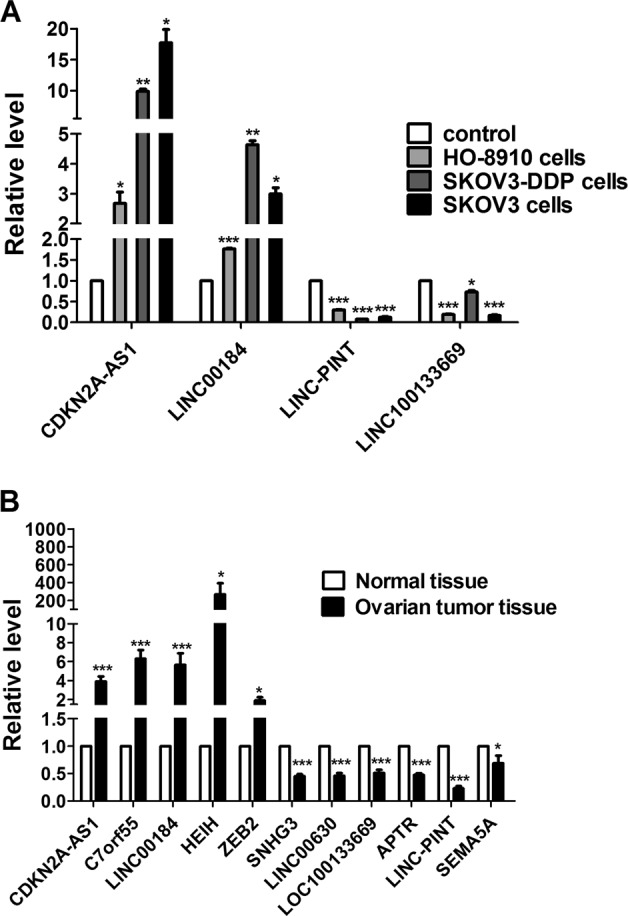
Relative expression of different lncRNAs in HO-8910, SKOV3, and SKOV3/DDP cell lines, normal ovary tissues, and OC tissues. Results of qPCR analyses. *P *<* 0.05; **P < 0.01; and ***P < 0.001 versus normal cells [a, *n* = 3; and b, *n* = 30]

Based on the microarray results of the 3527 lncRNAs, we identified four with the most significant differential expression between OC and normal ovary cells (*CDKN2A-AS1*, *LINC00184*, *LINC-PINT*, and *LOC100133669*). We performed real-time quantitative polymerase chain reaction (qPCR) analysis of the levels of these lncRNAs in the cell lines described, as well as a new ovary cancer cell line (HO-8910), in order to verify their expression. The results demonstrated that *CDKN2A-AS1 and LINC00184* were significantly upregulated, whereas *LINC-PINT* and *LOC100133669* were markedly decreased in HO-8910, SKOV3, and SKOV3/DDP cells as compared with levels in IOSE80 cells (Fig. 5a). To confirm these findings, ovary biopsy samples extracted during gynecological surgery from both OC patients and healthy individuals were used to evaluate differences in levels of the most dysregulated candidate lncRNAs, including *CDKN2A-AS1*, *C7orf55*, *LINC00184*, *HEIH*, *ZEB2*, *SNHG3*, *LINC00630*, *LOC100133669*, *APTR*, *LINC-PINT*, and *SEMA5A*. qPCR results indicated marked upregulation of *CDKN2A-AS1*, *C7orf55*, *LINC00184*, *HEIH*, and *ZEB2* expression, whereas that of *SNHG3*, *LINC00630*, *LOC100133669*, *APTR*, *LINC-PINT*, and *SEMA5A* was downregulated in OC tissues as compared with levels in normal ovary tissues (Fig. 5b).

### Construction of a lncRNA–mRNA co-expression network

**Fig. 6:**
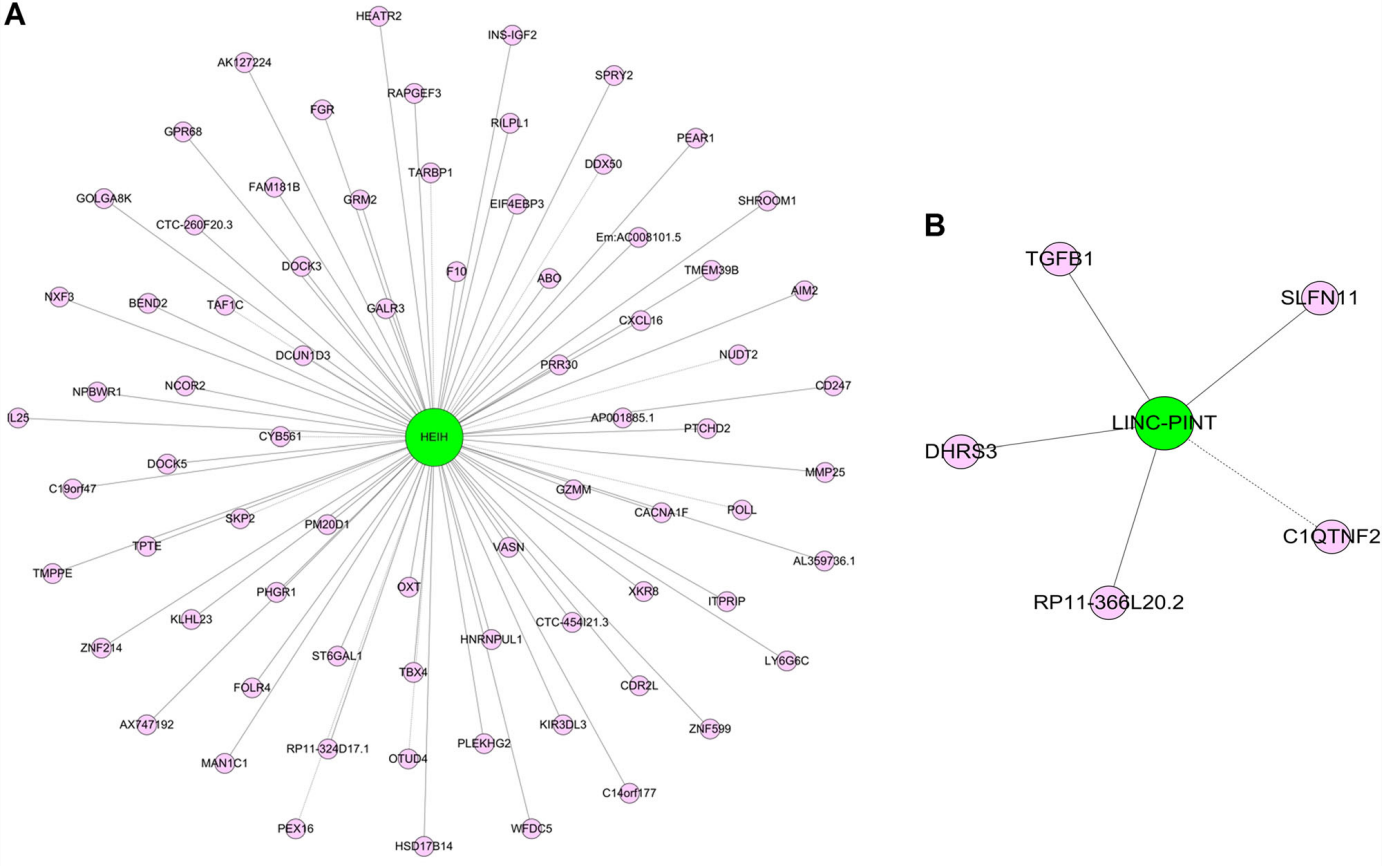
Construction of a lncRNA–mRNA co-expression network. Red nodes represent mRNAs, and green nodes represent lncRNAs. The lines between the red and green nodes represent interactions between mRNA and lncRNA. Solid lines indicate positive correlations, whereas a dashed line indicates a negative correlation

A lncRNA–mRNA co-expression network was constructed in order to identify mRNAs associated with specific lncRNAs. The two lncRNAs exhibiting the most significant differential expression (*HEIH* and *LINC-PINT*) were selected for building this co-expression network in order to evaluate potential associations with mRNAs. We selected 74 and 5 target mRNAs for *HEIH* and *LINC-PINT*, respectively, among which were those involved in important roles in cancer progression. For example, *S-phase kinase-associated protein 2* participates in the regulation of immune reactions, cell proliferation, and recruitment, and levels of *schlafen family member 11* are directly associated with chemotherapeutic and/or polyADP ribose polymerase-inhibitor sensitivity in a number of cancer types. In addition, upregulation of *MAN1A1* activates the unfolded protein response and might initiate metastasis. The cancer-specific roles of these mRNAs suggest the potential importance of *HEIH* and *LINC-PINT* in EOC (Fig. 6a, b).

### Suppression of lncRNA HEIH inhibits OC cell progression

**Fig. 7:**
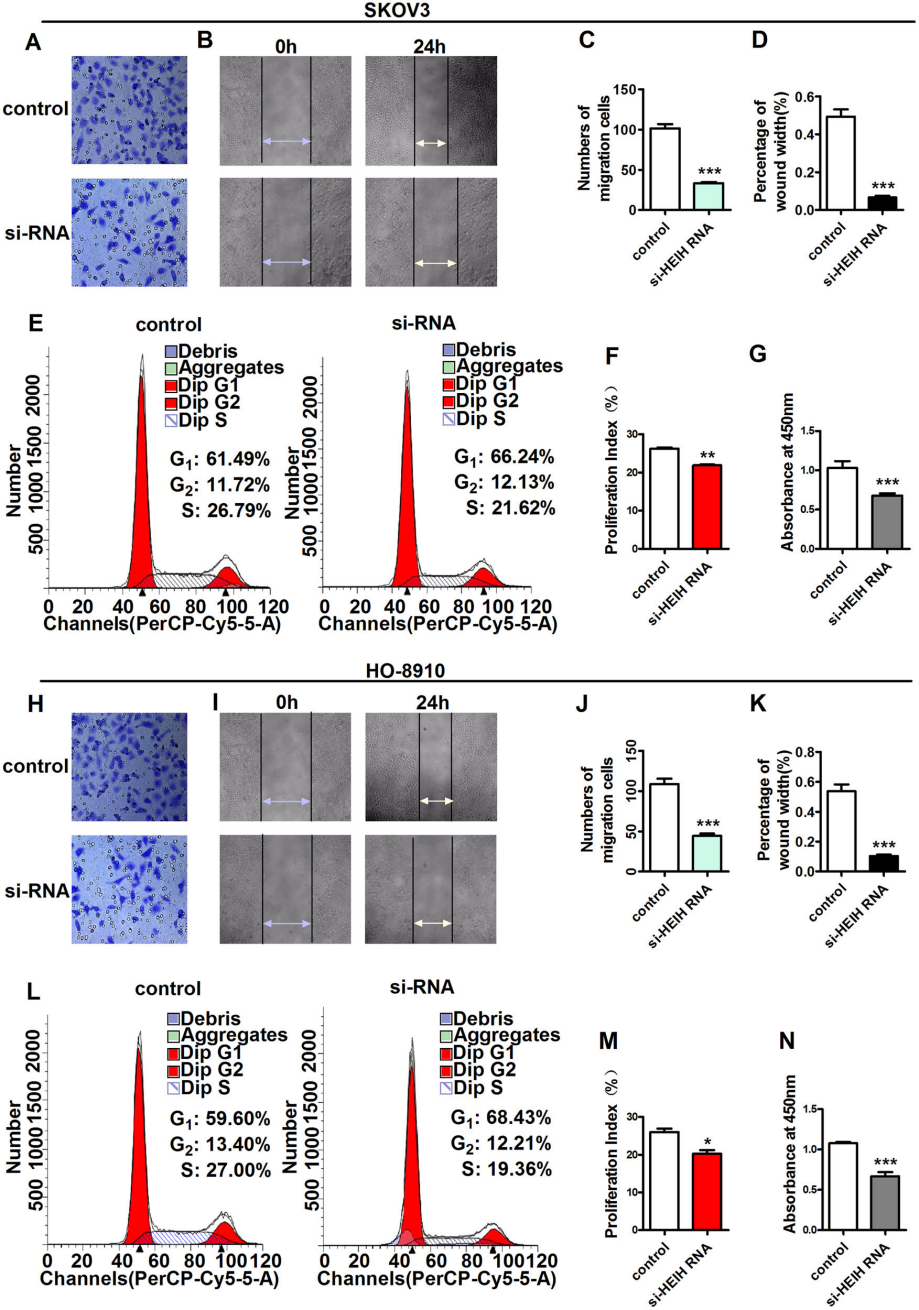
Changes in *HEIH* expression between SKOV3 and HO-8910 cells affect cell proliferation, cell cycle progression, and cell migration in vitro. SKOV3 and HO-8910 cells were transfected with si-*HEIH* for 24 h. **a, h** SKOV3 and HO-8910 invasion abilities according to the transwell migration assay. **b, i** Representative photomicrographs of wound healing in the presence of SKOV3 cells at 0 h and 24 h. **c, j** Number of SKOV3 and HO-8910 cells that invaded the substratum of the membrane per field of view. **d, k** Photomicrographs of wound healing in the presence of HO8910 at 0 h and 24 h. Arrows highlight the linear scratch/wound for each group of cells. The mean percentage of wound closure per group (*n* = 3). **e, f, l, m** Flow cytometric results showing cell cycle arrest in the si-*HEIH* group. **g, n** Proliferative ability according to the CCK-8 assay. Data represent the mean ± standard deviation. *P < 0.05; **P < 0.01; ***P < 0.001

To further investigate the function of *HEIH* in OC development, we transfected SKOV3 and HO-8910 cells with small-interfering (si)RNA targeting *HEIH* (si-HEIH), followed by assessment of cell proliferation, migration, and invasion. Both OC cell lines exhibited lower invasion rates relative to those of control cells, suggesting that *HEIH* silencing significantly reduced the invasive abilities of SKOV3 and HO-8910 cells (Fig. 7a, c, h, j). To evaluate the role for *HEIH* in cell migration, we performed a wound-healing assay, finding that *HEIH*-silenced cells exhibited slower wound-healing ability relative to controls (Fig. 7b, d, i, k). In addition, flow cytometry results showed a decreased number of cells in the S phase of the cell cycle (Fig. 7e, f, l, m). Moreover, Cell Counting Kit-8 (CCK-8) assays confirmed that *HEIH* silencing inhibited OC cell viability (Fig. 7g, n). These results suggested that *HEIH* siRNA might represent an efficacious method for suppressing tumor growth by inhibiting OC cell progression.

## Discussion

Numerous studies report that lncRNA dysregulation is associated with human cancers, including EOC^17^. However, studies of the expression profiles of lncRNAs in EOC or *cis*-resistant EOC, as well as those predicting correlations between lncRNA and malignant progression, are limited. In this study, we used microarrays to detect genome-wide lncRNA-expression patterns in SKOV3 cell lines and compared those with patterns in IOSE80 and SKOV3/DDP cell lines in order to elucidate their potential roles in EOC oncogenesis and progression. In addition, we evaluated correlated signaling pathways associated with several candidate lncRNAs identified from this analysis.

lncRNAs play important roles in the regulation of gene expression and tumor progression^18,19^, and their expression is closely associated with biological functions and tumor status^20^. Some lncRNAs exhibit tissue-specific, disease-specific, and developmental-stage-specific expression^20–22^. Previous studies identified *HOTAIR*^23^, *ANRIL*^24^, and *LSINCT5*^25^ as important regulators of OC. In the present study, we observed significant differential expression of some lncRNAs and mRNAs in SKOV3 and SKOV3/DDP cells as compared with IOSE80 cells, and that a higher number of genes exhibited differential expression in SKOV3/DDP cells relative to SKOV3 cells. Our results highlighted a unique lncRNA-expression profile in SKOV3 cells, with some of these lncRNAs possibly associated with chemotherapy resistance.

We found that most of the enriched GO terms and KEGG pathways associated with downregulated mRNAs were shared between SKOV3 and SKOV3/DDP cells. GO analysis revealed that differentially expressed lncRNAs were highly enriched in cellular response to IFN-γ, cellular metabolic processes, single-organism processes, and regulation of cellular biosynthetic processes. Moreover, the top predicted KEGG pathways were the p53 signaling pathway, cancer-related pathways, protein-processing pathways in the endoplasmic reticulum, and the MAPK signaling pathway, all of which are associated with EOC progression. Previous studies showed that lncRNAs play important roles in cancer by regulating associated signaling pathways, with *PINCR*^26^, *MIR31HG*^27^, and *ROR*^28^ affecting cancer-cell proliferation via the p53 signaling pathway, and *NNT-AS1*^29^ and *BANCR*^30^ influencing cancer-cell proliferation and invasion by regulating the MAPK signaling pathway. Our results indicated that dysregulated lncRNAs might regulate cancer-associated pathways and affect the pathogenic process of EOC. Moreover, the significantly enriched GO terms and KEGG pathways for upregulated RNAs between the SKOV3 and SKOV3/DDP cell lines differed considerably from those of the downregulated RNAs. Our findings suggested that lncRNAs potentially regulate chemotherapy resistance via the enhancement of particular signaling pathways.

Our findings indicated that the functions of most of the identified lncRNAs were ambiguous. We constructed a lncRNA–mRNA co-expression network in order to identify key lncRNAs associated with EOC. lncRNAs *HEIH* and *LINC-PINT* showed the highest number of neighbors in the network, suggesting potentially key roles in regulating gene expression and protein translation possibly involved in EOC progression. Previous studies reported that a conserved functional codependence between *LINC-PINT* and *polycomb repressive complex 2* counteracts gene activation by the early growth response-1 protein^31^, and that *HEIH* promotes melanoma-cell proliferation, migration, and invasion by inhibiting miR-200b/a/429 levels^32^. In addition, we found that part of the dysregulated lncRNAs in the SKOV3/DDP cell line showed a higher degree of significant differential expression relative to those in the SKOV3 cell line. Moreover, CCK-8 and flow cytometric analyses confirmed that *HEIH* silencing significantly reduced the proliferative capacity of OC cells, and wound-healing and transwell migration assays indicated key roles for *HEIH* in inhibiting OC cell migration and invasion.

In summary, our findings demonstrated that 8388 lncRNAs were differentially expressed in SKOV3 cell lines as compared with the IOSE80 cell line, and that multiple lncRNAs were identified as potentially involved in EOC-related signaling pathways. Our data provides a foundation for further investigations into the roles of *HEIH* in EOC oncogenesis and progression and will promote identification of novel therapeutic targets and diagnostic biomarkers in the future.

## Materials and methods

### Cell culture

IOSE80, SKOV3, and SKOV3/DDP cell lines were purchased from the American Type Culture Collection (Manassas, VA, USA). The HO-8910 human OC cell line was obtained from the Department of Obstetrics and Gynecology Laboratory of the Second Affiliated Hospital of Harbin (Heilongjiang, China), and Roswell Park Memorial Institute (RPMI)-1640 medium was purchased from Hyclone (South Logan, UT, USA). IOSE80, SKOV3, and SKOV3/DDP cells were cultured in Dulbecco’s modified Eagle medium supplemented with 10% fetal bovine serum (FBS). HO-8910 cells were cultured in RPMI-1640 medium supplemented with 10% FBS. All cell lines were cultured in a humidified incubator in an atmosphere of 5% CO_2_ at 37 °C.

### lncRNA and mRNA microarray

The Arraystar human lncRNA microarray (v.4.0; Arraystar, Rockville, MD, USA) was used for the global expression profiling of human lncRNAs and protein-coding mRNA transcripts in the EOC cell lines. Total RNA was amplified and labeled using a low-input QuickAmp labeling kit (One-Color; Agilent Technologies, Santa Clara, CA, USA). Labeled complementary (c)RNA was purified using an RNeasy mini kit (Qiagen, Hilden, Germany). The concentration and specific activity of the labeled cRNAs [pmol cyanine 3 (Cy3)/μg cRNA] were measured using a NanoDrop ND-1000 (Thermo Fisher Scientific, Waltham, MA, USA). Each slide was hybridized with 1.65 μg Cy3-labeled cRNA for 17 h at 65 °C in an Agilent hybridization oven using the Agilent gene-expression hybridization kit (No. 5188–5242; Agilent Technologies). The slides were washed in a slide-staining dish (Shandon; No. 121; Thermo Fisher Scientific) using a gene-expression wash buffer kit (Agilent Technologies) and scanned using an Agilent microarray scanner (G2565BA; Agilent Technologies). Feature-extraction software (v.11.0.1.1; Agilent Technologies) was used to extract data and analyze the acquired array images. Microarray data were saved in the Gene Expression Omnibus database (accession ID: GSE104776).

### RNA isolation and real-time qPCR

Total RNA was extracted from cells using TRIzol reagent (Thermo Fisher Scientific), with the amount of TRIzol reagent used based on the area of the culture dish (1 mL/10 cm^2^). Microarray data were validated by qPCR using SYBR Green master mix (Applied Biosystems, Foster City, CA, USA). *β-actin* was used as an internal control, and the 2^−ΔΔCT^ method was used for transcript quantification. Primers were designed using Primer 5 software (Premier Biosoft, Palo Alto, CA, USA), with the primer sequences listed in Table 1. All experiments were independently performed in triplicate.

**Table 1:** Primers used in this study

Gene	Forward (5′ → 3′)	Reverse (5′ → 3′)
*SNHG3*	GGAGACAGATTCGCAGTGGT	AAAGGAGGCATGAAATGCAC
*LINC00630*	GAGACGAAAGGACCCCAGAT	AGTAGCCCTGTCTCCAGCAA
*LOC100133669*	TACAGAGACCGAAGCGTCCT	TGTGAGGCCAGTGAAAAACA
*CDKN2A-AS1*	GAGGCCTGGTGAGCAAAATA	AAAGCCGTGTCTCAAGATCG
*C7orf55*	TTCGTGGGATAGGCAGAGAC	GCAGCTTGGAAATGAAGCTC
*LINC00184*	CCATTCATGATGTTGGGTCA	GGAAGGCTGGCAAGTAATGA
*HEIH*	CTGTGCTCGCATCACATACC	TGTGTGACCGATCAACTGGT
*ZEB2*	AGCCTCTGTAGATGGTCCAGTGA	AAGCGCTTGTAGCCCCGGTC
*SEMA5A*	CTACTTGCCGGGAAGGCGGC	CCCGGGATGAGCGACACTGG
*APTR*	ACACTGTTGCCGGTATCACA	GCTTGACAGCCTTCCACAAT
*LINC-PINT*	ACAAATCTACGTGCGCATCA	AGCAAGGCAGAGAAACTCCA

### Pathway analysis

Using the latest KEGG database (http://www.genome.jp/kegg), we performed pathway analyses for the differentially expressed mRNAs in order to determine significantly enriched biological pathways according to *P* < 0.05.

### GO analysis

We performed GO analysis in order to associate differentially expressed mRNAs with categories from the GO website (http://www.geneontology.org) according to *P* < 0.05, which denoted a significant GO term.

### lncRNA–mRNA co-expression network

We constructed a lncRNA–mRNA co-expression network in order to investigate the potential functions of the differentially expressed lncRNAs via interactions between mRNAs and lncRNAs. Networks were constructed using a previously described algorithm^33^. In the networks, red nodes represented mRNAs, and green nodes represented lncRNAs, with the lines between the red and green nodes representing interactions between mRNAs and lncRNAs. A solid line indicated a positive correlation, whereas a dashed line indicated a negative correlation.

### Transwell migration assay

A transwell migration assay was performed using Transwell inserts (BD Biosciences, San Jose, CA, USA) with an 8-μm pore-size filter. A total of 5 × 10^4^ cells in serum-free medium were seeded into the upper chamber of the insert pre-coated with Matrigel, and 500 μL of conditioned medium was added to the lower chamber. After a 24-h incubation, the cells were fixed with paraformaldehyde for 15 min and stained with 0.1% crystal violet, after which the cells on the top surface of the membrane were carefully removed, and the cells on the lower surface were examined by microscopy. Five random fields were photographed for counting purposes, and the average number of migrated cells was used as an indicator of migration capacity. All experiments were independently performed in triplicate.

### Cell-proliferation assay

Cell-proliferative capacity was measured using CCK-8 (Dojindo, Shanghai, China). Briefly, 2.5 × 10^3^ cells were seeded into wells after transfection and cultured for 24 h, after which 10 μL of CCK-8 reagent was added to each well, followed by incubation for 1 h at 37 °C. Optical density was measured using a microplate reader (Bio-Rad, Hercules, CA, USA) at a wavelength of 450 nm, and proliferation rates were calculated.

### Cell scratch test

SKOV3 and HO-8910 cells were seeded at 1 × 10^5^ cells/well on 1% gelatin-coated six-well plates (Corning, Apeldoorn, Netherlands). Confluent cells were serum-deprived for 16 h, and linear wounds were created in the monolayers by scratching with a sterile pipette tip. The monolayers were then washed with phosphate-buffered saline to remove floating cells, followed by addition of the conditioned medium. After a 24-h incubation, cell migration into the wound was assessed by microscopy. All experiments were independently performed in triplicate.

### Flow cytometry

We performed flow cytometric analysis in order to determine whether silencing *HEIH* inhibited the growth phase of SKOV3 and HO-8910 cells. SKOV3 and HO-8910 cells transfected with either negative control siRNA (si-NC) or si-*HEIH* were harvested at 48 h and resuspended at 1 × 10^6^ cells/mL, followed by the addition of Annexin V-FITC and propidium iodide (PI; BD Pharmingen, Franklin Lakes, NJ, USA) according to manufacturer instructions. Cells were fixed with 70% ice-cold ethanol and labeled with PI for growth-phase analysis. All experiments were conducted using a flow cytometer (FACScan; BD Biosciences).

### Statistical analysis

Data represent the mean ± standard error of the mean. Differences among groups were analyzed using one-away analysis of variance accompanied by a Newman–Keuls multiple-comparison test using GraphPad Prism software (v.5.0; GraphPad, San Diego, CA, USA). A two-tailed Student’s *t* test was used for comparison between two groups. A *P* < 0.05 was considered statistically significant.
